# Complete Pathological Response to Platinum-Based Neoadjuvant Chemotherapy in BRCA2-Associated Locally Advanced Pancreatic Cancer: A Case Report and Literature Review

**DOI:** 10.7759/cureus.43261

**Published:** 2023-08-10

**Authors:** Mohamed S Asiri, Alhomam Dabaliz, Mahdi Almutairi, Abdulaziz Almahbub, Mohammed Alharbi, Sarah Almeman, Saeed AlShieban, Tareq Alotaibi, Mohammed Algarni

**Affiliations:** 1 Medicine, College of Medicine, King Saud Bin Abdulaziz University for Health Sciences, Riyadh, SAU; 2 Medicine, King Abdullah International Medical Research Center, Riyadh, SAU; 3 Medicine, College of Medicine, Alfaisal University, Riyadh, SAU; 4 Pathology and Laboratory Medicine, King Abdulaziz Medical City, Riyadh, Riyadh, SAU; 5 Pathology and Laboratory Medicine, College of Medicine, King Saud Bin Abdulaziz University for Health Sciences, Riyadh, SAU; 6 Pathology and Laboratory Medicine, King Abdullah International Medical Research Center, Riyadh, SAU; 7 Medical Imaging, King Abdulaziz Medical City, Riyadh, Riyadh, SAU; 8 Oncology, King Abdulaziz Medical City, Riyadh, Riyadh, SAU; 9 Oncology, College of Medicine, King Saud Bin Abdulaziz University for Health Sciences, Riyadh, SAU; 10 Oncology, King Abdullah International Medical Research Center, Riyadh, SAU

**Keywords:** folfirinox, brca2, platinum-based therapy, locally advanced pancreatic adenocarcinoma, pancreatic adenocarcinoma

## Abstract

Pancreatic ductal adenocarcinoma (PDAC) is a lethal malignant disease and is considered the fourth leading cause of death among cancer patients in the United States. Mutations in the BRCA gene, which is a DNA repair gene, increase the risk of PDAC, and among all patients with PDAC, about 8%-10% have a BRCA2 mutation. The finding of gene mutations is associated with a better response to platinum-based chemotherapy. Here, we present a case of a 59-year-old male with a BRCA2 gene mutation who was diagnosed with locally advanced pancreatic cancer and had achieved a complete pathological response to the FOLFIRINOX (leucovorin calcium, fluorouracil, irinotecan hydrochloride, and oxaliplatin) regimen and Whipple procedure. We also present our literature findings on response types in BRCA2 PDAC patients, as well as consensus on the use of different therapies. The use of platinum-based chemotherapy with BRCA2 is highly recommended as the first-line treatment. Most PDAC patients remain untested for BRCA2 mutation even though their genetic status influences the selection of drug regimens. Thus, we recommend genetic testing for everyone with PDAC.

## Introduction

Pancreatic ductal adenocarcinoma (PDAC) is considered one of the deadliest malignancies worldwide, associated with poor prognosis at all clinical stages [1-2]. According to the Surveillance, Epidemiology, and End Results Program (SEER), it is the fourth leading cause of cancer-related mortality in the United States and has a predilection for older individuals. Breast cancer genes BRCA1 and BRCA2 have an important role in DNA repair, specifically in the homologous recombination pathway [3]. Mutations in these genes are a well-known factor that increases the risk for many types of cancer, including pancreatic cancer whose incidence has been reported to increase three- to fivefold in patients with BRCA1 or BRCA2 mutations [4]. Algarni et al. estimated a germline mutation prevalence of 8.1% for pancreatic cancer in the Saudi population, with BRCA2 mutation representing nearly half [5]. BRCA mutations are also important for the prognosis of the disease; a cohort study on patients with metastatic PDAC with BRCA gene mutations who were treated with platinum-based chemotherapy (PtCh) agents showed a significantly longer survival time and higher objective response rate than in those treated with other agents [1,4,6-8]. These agents work by inducing DNA damage, which can be extremely deadly in a cell that has lost its DNA repair mechanisms, like cells with BRCA mutations [9]. Although PtCh should be effective in all cases with mutations that damage the homologous recombination pathway of DNA repair, it was found that only patients with either BRCA or PALB2 (another gene encoding for a protein used in repairing double-strand breaks) mutations showed increased sensitivity to them, while other mutations did not show any change in response to those agents [2,7-8]. On the other hand, however, PtCh agents did not show any increased effectiveness in patients without those mutations compared to non-PtCh agents [2]. With this in mind, we present a case of locally advanced pancreatic cancer (LAPC) with BRCA2 in a patient who responded exceptionally well to neoadjuvant PtCh and Whipple procedure, along with a review of the literature for the consensus on how to treat this subset of cancer.

## Case presentation

**Figure 1:**
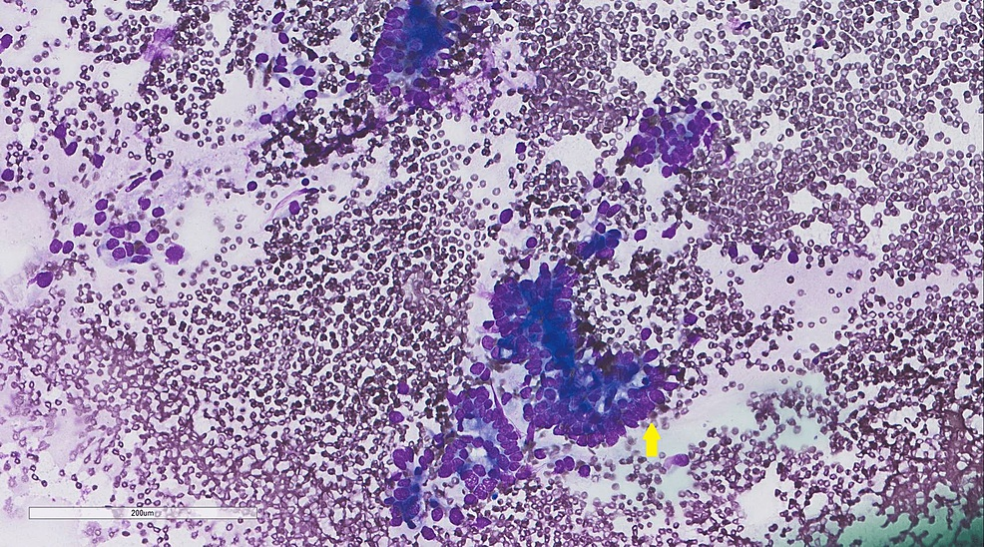
Diff-Quik stain cytology Endoscopic ultrasound-guided fine needle aspiration showing a group of atypical epithelial cells with crowded nuclei and mild polymorphism (arrow).

**Figure 2:**
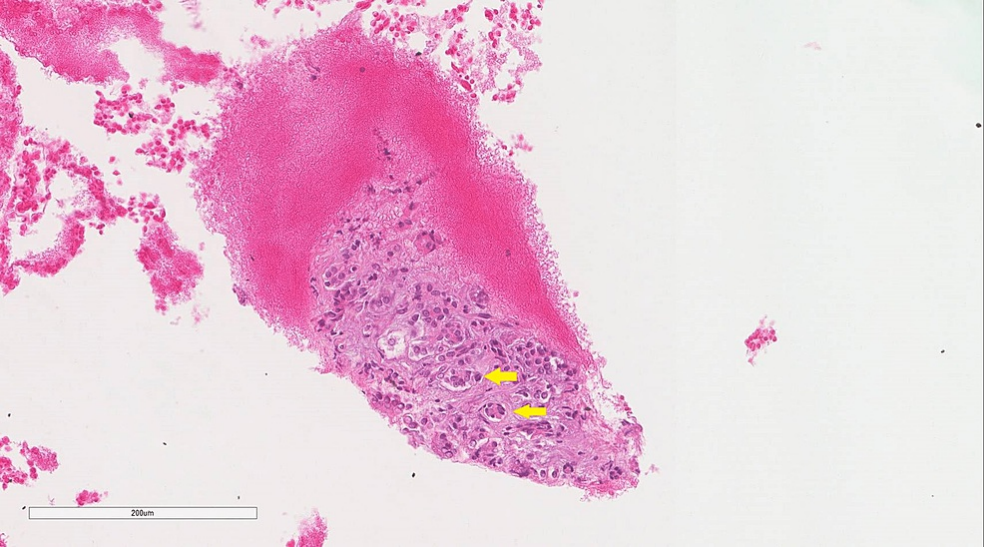
Cell block preparation (hematoxylin and eosin stain, magnification 20x) A group of atypical epithelial cells with crowded nuclei and mild polymorphism (arrows).

**Figure 3:**
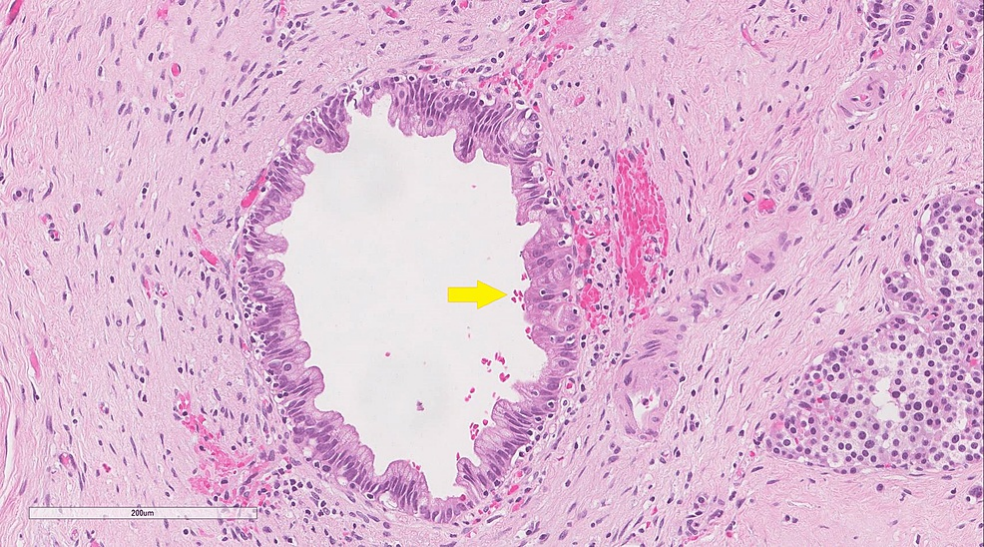
A low-grade pancreatic intraepithelial neoplasia (arrow) Hematoxylin and eosin stain (magnification 40x)

A 59-year-old man came to the adult oncology clinic for worsening right upper quadrant abdominal pain; the pain was present for the past three months and was progressing in nature. It was not associated with food consumption, blood in stools, or vomiting. The patient had a 20 kg weight loss within the past three months and recent scleral jaundice. Urine and bowel changes were unremarkable. He came from a nearby secondary care center where multiple investigations and procedures had been done. Notably, magnetic resonance cholangiopancreatography (MRCP) showed intra- and extrahepatic biliary dilation of 1 cm, upon which endoscopic retrograde cholangiopancreatography (ERCP) with an endoscopic ultrasound biopsy was performed. Fine needle aspiration (FNA) showed atypia with likely malignancy. He was transferred to our tertiary care center for further assessment. The patient was a known diabetic and was not on any medications. Upon further questioning, no history of pancreatic, breast, ovarian, or prostate cancer was found in his family. His physical examination was notable for scleral icterus; the remaining examination was unremarkable. Initial labs showed an elevated total bilirubin level at 2.1 mg/dL and a CA 19-9 level of 2602 U/mL. Endoscopic ultrasound-guided FNA (EUS-FNA) was performed, which yielded a few groups of atypical epithelial cells. These atypical epithelial cells showed enlarged, hyperchromatic, crowded nuclei and mild polymorphism seen in the Diff-Quik stain (Figure 1) and cell block preparation (Figure 2). A low-grade pancreatic intra-epithelial neoplasia (PanIN) was also identified (Figure 3).

**Figure 4:**
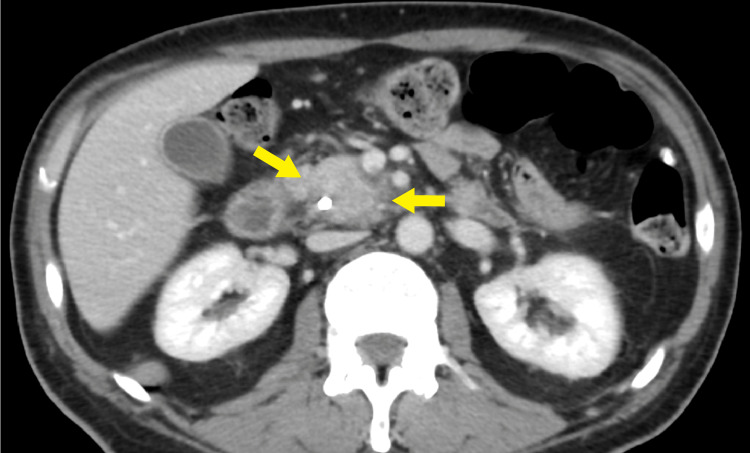
Axial CECT image showing the infiltrative hypodense pancreatic head lesion The lesion is abutting the SMA by 180 degrees. There is associated abutment and irregularity of the portal vein near the SMV, suggestive of pancreatic adenocarcinoma. The CBD stent is present. CECT: contrast-enhanced computed tomography; SMV: superior mesenteric vein; SMA: superior mesenteric artery; CBD: common bile duct

**Figure 5:**
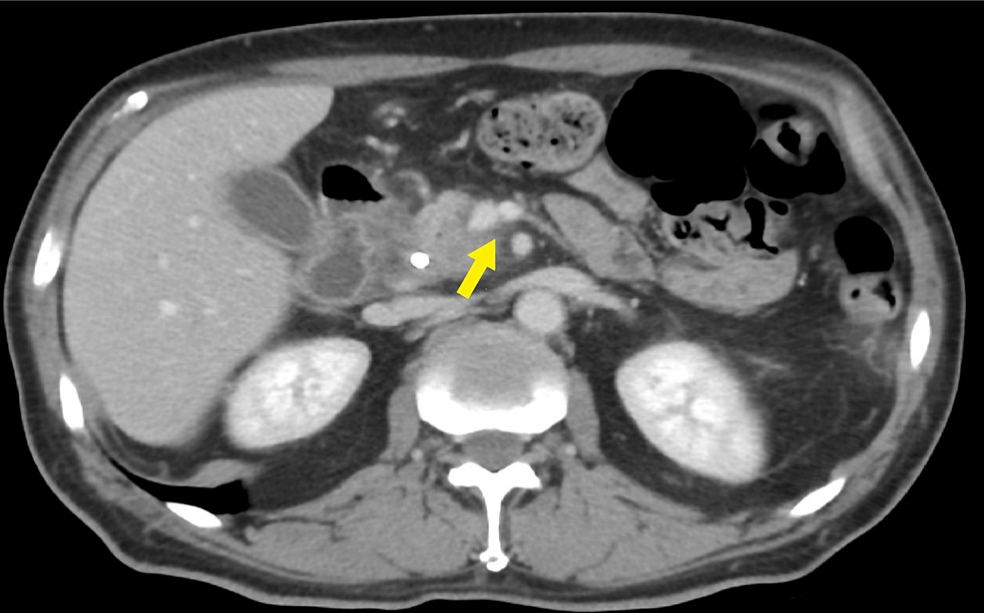
Another axial CECT image showing the infiltrative hypodense pancreatic head lesion CECT: contrast-enhanced computed tomography

**Figure 6:**
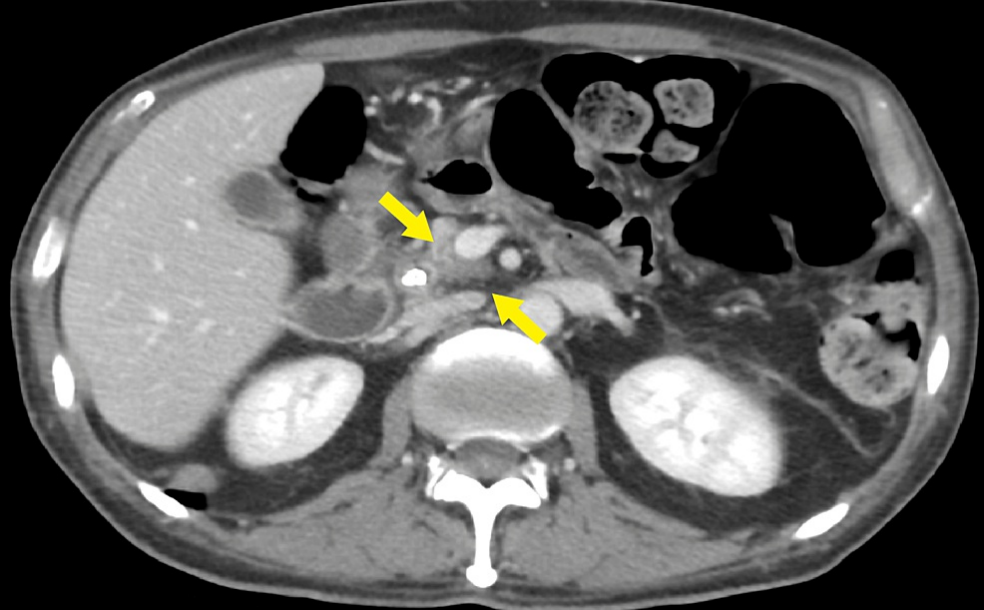
Post-neoadjuvant therapy axial CECT image shows significant improvement in the size of the pancreatic head adenocarcinoma There is persistent abutment of the portal vein/SMV confluence. The SMA is spared. CECT: contrast-enhanced computed tomography; SMV: superior mesenteric vein; SMA: superior mesenteric artery

The case was discussed by a multidisciplinary oncology team, and taking into account the clinical, pathological, and radiological findings (Figures 4-5), they recommended starting him on neoadjuvant chemotherapy and re-evaluating after chemotherapy regarding resectability. The current policy at our institute is to utilize neoadjuvant protocols, especially for gastrointestinal tumors. The patient was started on a neoadjuvant six-cycle course of FOLFIRINOX regimen (leucovorin calcium, fluorouracil, irinotecan hydrochloride, and oxaliplatin) with standard dosing and scheduling that was tolerable with no adverse events other than fatigue, nausea, and vomiting. Genetic testing was ordered as all newly diagnosed pancreatic cancer cases at our institute undergo genetic testing regardless of the family history. The result was consistent with a genetic diagnosis of autosomal dominant hereditary breast and ovarian cancer syndrome. CT imaging of the lesion prior to chemotherapy (Figures 4-5) and after the last cycle by a week (Figure 6) showcased differences in the size of the tumor with subsequent improvement in duct dilation. Surgery was scheduled three months later for the Whipple procedure. It was performed using a right-sided chevron incision. The duodenum was accessed fully with Kocherization, removing all surrounding tissues down to the inferior vena cava and aorta. After the proximal jejunum was mobilized and the lesser sac was entered, the middle colic was followed that helped identify the superior mesenteric vein, and a plane was established behind the pancreatic neck. The remainder of the procedure went on smoothly, with roughly 250 mL of blood loss, and lasted six hours. The patient was discharged after two days and was booked for follow-up for imaging and labs with the oncology clinic.

**Figure 7:**
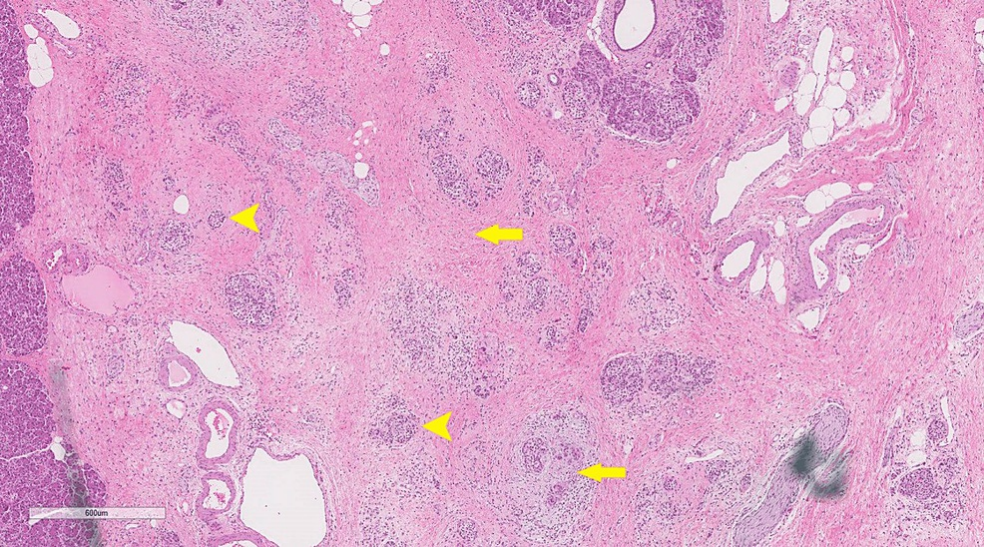
Resected tumor bed (hematoxylin and eosin stain, magnification 4x) Pancreatic parenchymal atrophy (arrowheads) and intralobular and interlobular fibrosis (arrows), with complete treatment response.

**Figure 8:**
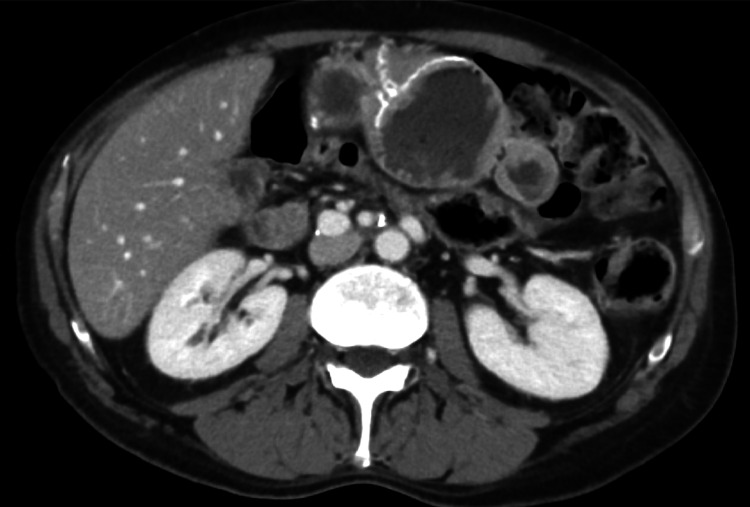
Axial CECT image demonstrating tissue bed post-Whipple procedure There are scar changes without local reoccurrence. CECT: contrast-enhanced computed tomography

Macroscopically, the pancreas measured 7.0 × 5.0 × 3.0 cm. Serial sections revealed an ill-defined fibrotic lesion located near the uncinate process measuring 1.3 x 1.0 x 0.7 cm. Microscopically, the pancreas showed a tumor bed with intralobular and interlobular fibrosis with atrophic pancreatic acini (Figure 7), with no tumor cells seen indicating a complete treatment response. No lymph nodes were seen and margins were negative. The patient had been feeling better months afterward, and pathology (Figure 7), serial imaging (Figure 8), and lab testing showed no evidence of recurrence. The patient has been followed up for 18 months as of now with serial CT imaging and CA 19.9 levels showing no recurrence thus far.

## Discussion

PDAC is commonly diagnosed at an advanced stage due to its insidious clinical presentation of abdominal pain and weight loss [1]. In many cases, the tumor is diagnosed with concurrent metastasis and advanced into the surrounding anatomical organs. Currently, the most used PtCh regimen is FOLFIRINOX that comprises leucovorin calcium, fluorouracil, irinotecan hydrochloride, and oxaliplatin [7]. However, other regimens exist and are used, such as FOLFOX and oxaliplatin plus gemcitabine [7-8]. A retrospective study done by Wattenberg et al. suggests that there is no significant difference in objective response rates between these different regimens in patients with BRCA mutations; however, FOLFIRINOX was the only regimen that showed any response (albeit small) in patients without BRCA mutations [8]. In a meta-analysis by Rebelatto et al., patients with clinical stage III-IV PDAC on PtCh agents had lower mortality compared to those on non-PtCh agents (62.3% vs. 87.5%) and an overall survival time 10 months longer as well (23.7 months vs. 12.2 months) [1]. Another cohort study performed by Golan et al. showed an overall survival in stage III-IV patients on PtCh agents of 22 months compared to 9 months for patients on non-PtCh agents [6]. This same cohort also showed that both BRCA1 and BRCA2 mutations had an equally positive prognosis, even though BRCA2 mutations are more common in PDAC patients; this is also supported by other cohort studies [6-7]. It is important to note that the highly favorable results of using PtCh agents in BRCA mutation-positive PDAC are only very noticeable when used as a first-line agent, as the Wattenberg et al. study showed a sizeable difference between the progression-free survival of patients started on PtCh as first-line versus second-line or later (21.1 months vs. 2.5 months) [8].

PtCh is not only effective in the setting of medical management, as a retrospective study done by Yu et al. showed a significantly increased median overall survival and time to progression in BRCA- or PALB2-positive patients taking PtCh perioperatively (adjuvant or neoadjuvant) compared to mutation-negative patients taking PtCh [2]. However, PtCh agents are not the only effective treatment in BRCA-positive patients; a case reported by Pimenta et al. discussed a PDAC patient who showed a significant response to olaparib, a poly(adenosine diphosphate-ribose) polymerase inhibitor (PARPi), as a maintenance drug [10]. Those drugs inhibit a family of proteins responsible for repairing single-stranded DNA breaks arising during replication [11] and have been used before on patients with breast and ovarian cancer carrying a BRCA mutation and showed a good response [12-13]. Although PARPi agents have shown great potential as a maintenance drug, cases of BRCA2 pathogenic mutation-positive patients developing resistance to them have been reported. Many mechanisms for this resistance have been proposed, like functional reversion of BRCA2, defects in drug transport, or drug metabolism [14]. Most of these cases were seen in BRCA2-positive ovarian tumors treated with PtCh and PARPis. What is very peculiar about some of these cases is that they also developed a resistance to PtCh alongside PARPis [15-17], while others showed resistance only to PARPis and retained sensitivity to PtCh [18]. To our knowledge, there is only one case of PARPi resistance developed in a PDAC patient because of BRCA2 reversion mutation as reported by Pishvaian et al. [14]. Many reports highlight the potential benefit of using a combination of agents that target the deficient homologous recombination pathway in BRCA mutated cells including PtCh agents, cyclophosphamide, temozolomide, and PARPis [6,19]. Genetic studies may also be necessary to specify the zygosity status in the patient, as a cohort study done by Momtaz et al. demonstrated a decreased overall survival in monoallelic BRCA mutation patients compared to biallelic patients (10 months vs. 25 months, both stage IV); this also applied to patients on PtCh agents and PARPis [20]. However, these results may be misleading as the subgroup with monoallelic BRCA mutations had a much smaller number of patients [20]. Still, these findings may show merit as they were similar to Golan et al.’s findings [6].

Another drug class that has shown a possible benefit in patients with BRCA mutations is that of immunotherapy drugs [21-22]. Examples of these drugs include anti-PD1 (programmed death inhibitor) and anti-CTLA4 (cytotoxic T-lymphocyte associated protein 4) therapy [19]. However, in Momtaz et al.’s cohort study, only 1 out of the 10 patients who received immunotherapy showed a partial response; notably, this patient was the only one receiving a combination of anti-PD1 and anti-CTLA4 as their maintenance therapy. Ultimately, a multidisciplinary approach combining surgical, medical, and radiotherapy is thought to exemplify the optimal approach that can give excellent outcomes in some patients with LADC [23].

Recent studies have shown that a majority of PDAC patients with BRCA mutations are not genetically tested because they do not meet the previous criteria for BRCA genetic testing set by the National Comprehensive Cancer Network (NCCN) nor the criteria set by the Ontario Ministry of Health and Long-Term Care [24-25]. This has led the NCCN and the American Society of Clinical Oncology to amend their guidelines and recommend performing a genetic risk assessment for all patients with pancreatic cancer regardless of age [1,8]. This decision is based on the clearly better prognosis associated with providing patients with BRCA mutations with personalized treatment regimens. In another study by Emelyanova et al., characteristics of patients with PDAC were analyzed to identify the best factors that help guide the decision to perform genetic testing, and among all the characteristics studied, age <55 years and a personal history of ovarian, breast, pancreatic, or prostate cancer were the most highly associated with positive BRCA mutations [7]. A literature summary of all published cases, either as part of larger cohorts and trials or as individual case reports in the literature, categorized based on response percentages and regimens used is illustrated in Table 1.

**Table 1:** Overall PDAC treatment and clinical outcomes in BRCA-mutation-positive patients as found in the literature mPFS: median progression-free survival; mOS: median overall survival; PtCh: platinum chemotherapy; RAMPS: radical antegrade modular pancreatosplenectomy; FOLFIRINOX: leucovorin calcium (folinic acid), fluorouracil, irinotecan hydrochloride, and oxaliplatin; FOLFIRI: leucovorin calcium (folinic acid), fluorouracil, and irinotecan hydrochloride; FOLFOX: leucovorin calcium (folinic acid), fluorouracil, and oxaliplatin; PARPi: poly(ADP-ribose) polymerase inhibitor *All patients were either BRCA or PALB positive **Controlled disease = partial, complete, or stable

Reference	No. of patients	Treatment	Clinical course/outcome
POLO trial [26]	90	PtCh (not specified), olaparib	mPFS = 7.4 months, placebo mPFS = 3.8 months
	2		Complete remission
Sutton et al. [23]	1	RAMPS + FOLFIRINOX, ablative hypofractionated radiotherapy, olaparib	Partial response, initial treatment with RAMPS + FOLFIRINOX resulted in remission for 31 months; a new mass was discovered that was ablated with radiotherapy; currently asymptomatic on maintenance, olaparib 55 months post-RAMPS
Momtaz et al. [20]	46	PtCh (not specified)	Partial response, stage IV, first-line PtCh for 9 months
	6		Stable disease, stage IV, first-line PtCh for 9 months
	7		Partial response, stage IV, first-line PtCh for 9 months
	7		Partial response, stage IV, second-line PtCh for 11 months
	1		Stable disease, stage IV, second-line PtCh for 11 months
	10	PtCh (not specified), olaparib	Partial response, stage IV, maintenance PARPi post stable disease
	12		Stable disease, stage IV, maintenance PARPi post stable disease
	2		Stage IV, therapy PARPi post disease progression gave partial response
	7		Stage IV, therapy PARPi post disease progression gave stable disease
Pimenta et al. [10]	1	FOLFIRINOX, FOLFIRI, olaparib	13 cycles of FOLFIRINOX resulted in toxicity; stopped chemotherapy for 16 months leading to relapse; started 8 cycles of FOLIRI, disease still progressed; 3 cycles of olaparib led to a significant response; stable disease 6 cycles later
Golan et al. [6]	18	Gemcitabine + cisplatin	Stage III-IV, received PtCh, mOS of 22 months
	1	Gemcitabine + oxaliplatin	
	3	FOLFIRINOX	
Emelyanova et al.* [7]	10	FOLFIRINOX	6 had stable disease and 4 had no progression, mPFS = 12.7 months
	2	Gemcitabine + cis/oxaliplatin	No objective response, mPFS = 4.4 months
Wattenberg et al.* [8]	6	FOLFIRINOX	Partial response
	4	FOLFOX	Partial response
	4	Gemcitabine + cisplatin	Partial response
Yu et al.* [2]	32	Curative intent surgical resection, 9 neoadjuvant treatments, 11 perioperative PtCh, 3 FOLFIRINOX	12 had recurrence and received palliative PtCh, mOS of 46.6 months, mPFS of 13.4 months
O’Reilly et al.* [27]	27	Cisplatin, gemcitabine, veliparib	Controlled disease**; were given cisplatin + gemcitabine + veliparib combination, mPFS of 10.1 months, mOS of 15.5 months, 7 alive at data cutoff
	18	Cisplatin, gemcitabine	Controlled disease**; were given cisplatin + gemcitabine combination, mPFS of 9.7 months, mOS of 16.4 months, 2 alive at data cutoff

PARPis have garnered major interest as a possible first-line or maintenance treatment for BRCA mutation PDAC recently, with two of the most significant clinical trials investigating it: those by Golan et al. and O’Reilly et al. [26-27]. The POLO trial by Golan et al. was scrutinized for a plethora of reasons. Nishikawa and colleagues argued the poor design of the trial due to the primary focus on progression-free survival, which did not correlate with overall survival, and the suboptimal control arm that forced patients to halt chemotherapy when they were randomized to a placebo [28]. O’Reilly’s trial was a randomized, multicentric, phase II clinical trial designed to compare and evaluate the overall response rate (ORR) for the combination of cisplatin, gemcitabine, and veliparib to the ORR for cisplatin and gemcitabine alone. While the addition of veliparib did not significantly increase the ORR of the group that took it, the ORR of both groups was found to be impressively high leading the authors to recommend cisplatin and gemcitabine as a standard of care for BRCA-mutated/PALB2+ PDAC. However, this recommendation came with its issues such as biases, heterogeneity, differing standards of regimens used, and the fact that the regimen recommended by the trial has not shown significantly improved overall survival.

## Conclusions

The existence of BRCA gene mutation influences chemotherapy response as PDAC patients with BRCA mutations have more sensitivity to PtCh that works by inducing DNA damage resulting in tumor cell death. Due to noticeable differences in patients with BRCA mutation versus non-BRCA patients in regard to response to chemotherapy as adjuvant or neoadjuvant, genetic testing plays a significant role in response as well as determining which drugs are most effective. For this part, many BRCA mutation patients who are diagnosed with PDAC remain untested as they do not fit the criteria for genetic testing. Thus, both the National Comprehensive Cancer Network and the American Society of Clinical Oncology have recommended genetic testing for all pancreatic cancer patients regardless of age. Based on our findings, we encourage genetic testing for all patients since the percentage of BRCA gene mutation ranges from 8% to 10% among all pancreatic cancer patients; those patients have better outcomes and improved survival time when the status of their BRCA and other gene mutations is known and aids the selection of chemotherapeutic drugs.
